# Resignations

**Published:** 1858-09

**Authors:** 


### RESIGNATIONS.

Prof. S. G. Armor has resigned the Chair of Pathology and Clinical Medicine in the Missouri Medical College. Dr. McMartin, of St. Louis, takes his place.

Prof. Merrill has resigned the Chair of Theory and Practice of Medicine in Memphis Medical College, and John Pitman, M.D., has been elected to fill his place. Prof. Wright has resigned the Chair of Physiology and Pathological Anatomy in the same institution, and W. J. Tuck, M.D., has been elected to the place.

